# How do red blood cells know when to die?

**DOI:** 10.1098/rsos.160850

**Published:** 2017-04-05

**Authors:** Clemente Fernandez Arias, Cristina Fernandez Arias

**Affiliations:** 1 Departamento de Matemática Aplicada, Universidad Complutense de Madrid, Spain; 2 Grupo Interdisciplinar de Sistemas Complejos, Madrid, Spain; 3 HIV and Malaria Vaccine Program, Aaron Diamond AIDS Research Center, Affiliate of The Rockefeller University, New York, NY, USA

**Keywords:** red blood cell homeostasis, oxygen homeostasis, neocytolysis, erythropoietin, CD47, phosphatidylserine

## Abstract

Human red blood cells (RBCs) are normally phagocytized by macrophages of splenic and hepatic sinusoids at 120 days of age. The destruction of RBCs is ultimately controlled by antagonist effects of phosphatidylserine (PS) and CD47 on the phagocytic activity of macrophages. In this work, we introduce a conceptual model that explains RBC lifespan as a consequence of the dynamics of these molecules. Specifically, we suggest that PS and CD47 define a molecular algorithm that sets the timing of RBC phagocytosis. We show that significant changes in RBC lifespan described in the literature can be explained as alternative outcomes of this algorithm when it is executed in different conditions of oxygen availability. The theoretical model introduced here provides a unified framework to understand a variety of empirical observations regarding RBC biology. It also highlights the role of RBC lifespan as a key element of RBC homeostasis.

## 1. Introduction

The population of red blood cells (RBCs) in the organism must remain within definite limits in order to ensure the oxygenation of body tissues and to maintain adequate values of blood pressure and viscosity. This is achieved by means of homeostatic mechanisms that control the ratio between cell production and destruction and compensate any unbalance between oxygen supply and demand by increasing or reducing the number of circulating RBCs [1,2].

The formation of new RBCs is controlled by erythropoietin (Epo), a hormone produced by fibroblasts of peritubular capillaries in the kidney that induces proliferation and differentiation of erythroid precursor cells in the bone marrow [3]. On the other hand, RBCs are removed by macrophages of the mononuclear phagocytic system (MPS) when passing through the splenic and hepatic sinusoids. Macrophages identify and phagocytize RBCs that have attained a critical age (120 days in humans and 60 days in mice) in a process known as erythrophagocytosis [4–6].

In hypoxia, fibroblasts increase the release of Epo, thus accelerating the production of new cells and boosting the population of RBCs [7,8]. Conversely, if oxygen levels rise above physiological needs (e.g. in acclimation to higher partial pressure of oxygen after descent to sea level from high altitudes), fibroblasts lower the production of Epo and the population of RBCs shrinks to a new equilibrium size [3,9,10]. Excess of oxygen supply also entails an increase in the rate of cell destruction caused by neocytolysis, a homeostatic mechanism that entails the selective removal of RBCs of only 10 or 11 days of age, and contributes to the rapid reduction of the number of cells [11–14].

The switch from 120 days to 11 days of duration in response to environmental factors indicates that lifespan is not a fixed, intrinsic feature of RBCs. This point is further evidenced by the fact that RBCs live around 40 days less in newborn humans than in adults [15]. Even if the mechanisms that regulate changes in RBC lifespan remain obscure, it is widely assumed that RBC ageing and death are ultimately caused by oxidative stress (OS) [16–18]. The continuous exposure to highly reactive oxygen radicals deteriorates the membrane and cytoplasm of the RBC, which may eventually compromise its function [19]. In fact, higher sensitivities to OS correlate with shorter lifespans [20]. This observation has been interpreted as evidence of an active mechanism that would set RBC lifespan by fine-tuning the expression of genes that confer resistance to OS in erythroid precursors [16,20]. From this approach, human RBCs would be genetically configured to show signs of OS-driven senescence around the age of 120 days. Macrophages of the MPS would then identify aged RBCs by means of these signs [21].

In our opinion, the explanation of RBC lifespan as determined exclusively by OS is incomplete. For one thing, not all aged RBCs show the typical signs of severe OS-derived damage, such as cell shrinkage and membrane blebbing [22,23]. As a matter of fact, defective RBCs of any age are not destroyed by erythrophagocytosis, but through an alternative mechanism known as eryptosis [24,25]. This suggests that normal and damaged RBCs follow different phagocytosis pathways. On the other hand, the 10-fold decrease in RBC lifespan during neocytolysis would require a substantial reduction in the resistance of RBCs to oxidative damage. This would multiply the risk of RBC malfunction, a feature that seems unlikely for a physiological homeostatic mechanism. Alternatively, neocytolysis and erythrophagocytosis might be driven by different mechanisms [3,11], implying that some aspects of RBC lifespan cannot be explained by OS alone.

We postulate in this work that OS should not be considered as the key determinant of RBC lifespan, even if it causes the destruction of a fraction of circulating cells, and certainly imposes an upper boundary to the potential duration of RBCs in the blood. We suggest that lifespan is set by means of a molecular algorithm that controls cell-to-cell interactions between RBCs and macrophages of the MPS. We will show that such an algorithm could allow to fine-tune RBC lifespan in a variety of ways, thus providing a flexible system to adapt the number of cells to the demand of oxygen in the tissues.

The view of RBC lifespan introduced here frames a theoretical foundation in which to integrate different observations regarding RBC biology, such as erythrophagocytosis, neocytolysis and the seemingly paradoxical presence of auto-antibodies against host RBCs in the organism. In particular, we will show that these phenomena emerge as alternative outcomes of the same mechanisms working under different conditions of oxygen availability.

## 2. A conceptual model of red blood cell lifespan determination

**Figure 1.**
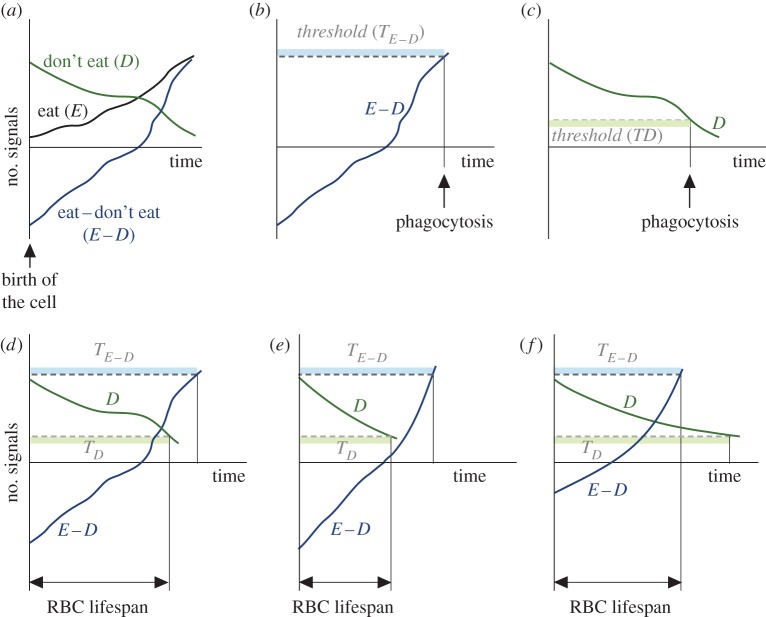
Rationale of the conceptual model of RBC lifespan determination. (*a*) Time evolution of membrane signals in a RBC according to empirical evidence (see points E1 and E2). (*b*) A RBC is phagocytized when the difference between ‘eat-me’ and ‘don’t-eat-me’ signals in its membrane attains a critical threshold (evidence E3). (*c*) An RBC can also be phagocytized if its level of ‘don’t-eat-me’ signals falls below a critical threshold (E4). (*d*) The conditions triggering RBC phagocytosis are mutually exclusive. In this example, the phagocytosis of the RBC occurs because the expression of ‘don’t-eat-me’ signals falls below a critical threshold (condition E4). (*e*,*f*) Different dynamics of membrane signals result in different lifespans (*e*) or in the RBC being phagocytized because it fulfils condition E3 before condition E4 (*f*).

The phagocytosis of RBCs by macrophages of the MPS is known to be mediated by phosphatidylserine (PS) and CD47 [26–31]. PS and CD47 have been labelled as ‘eat-me’ and ‘don’t-eat-me’ signals, owing to their pro- and anti-phagocytic effects, respectively [32,33]. Available empirical evidence concerning the dynamics of PS and CD47 expression in the membrane of RBCs can be summarized as follows:

(E1) PS is confined to the inner layer of the cell membrane in newly formed RBCs, so it is invisible for macrophages. Such membrane asymmetry is progressively lost in ageing RBCs, which increases PS exposure in the cell surface [34–36]. Therefore, pro-phagocytic effect of PS intensifies with the age of the RBC (figure 1*a*).
(E2) Conversely, the anti-phagocytic activity of CD47 is higher at the birth of the RBC [37,38]. Progressively lower expression of the protein or conformational changes in its spatial structure diminish its activity as a phagocytosis inhibitor as the cell ages [39] (figure 1*a*).
(E3) The effects of PS and CD47 cancel out each other [40], so that the net balance between PS and CD47 in an RBC determines whether or not it is destroyed by macrophages [22]. From points E1 and E2, it follows that ‘don’t-eat-me’ signals offset ‘eat-me’ signals in the membrane of young RBCs, preventing their phagocytosis. The difference between ‘eat-me’ and ‘don’t-eat-me’ signals grows in ageing RBCs until it reaches a critical threshold that elicits their destruction by macrophages of the MPS [40,41] (figure 1*b*).
(E4) It has also been observed that RBCs with sufficiently low levels of CD47 are also phagocytized regardless of the amount of PS present in their surface [42,43]. In this case, young RBCs are not destroyed because of the anti-phagocytic effect of CD47 (evidence E2). Owing to progressive loss of CD47 activity in ageing RBCs ‘don’t-eat-me’ signals eventually fall below a certain level that prompts the phagocytosis of the cell (figure 1*c*).

The conditions that trigger RBC phagocytosis (points E3 and E4) seem to be simultaneously fulfilled by ageing RBCs. However, since any particular RBC can only be phagocytized once, both conditions are in fact mutually exclusive. Only the first of the thresholds to be reached determines the lifespan of the RBC (figure 1*d*–*f*). On the other hand, both conditions seem to accomplish the same purpose, since both foster the phagocytosis of aged RBCs and the survival of young cells. This raises the question of why two apparently redundant pathways of RBC removal exist.

In order to address this issue, we begin by remarking that CD47 and PS also play a major role in the control of the phagocytosis of other cell types by macrophages [44–46]. Specifically, CD47 is broadly expressed in the host and absent in foreign cells [29,47], while PS is confined to the membrane of apoptotic host cells [48,40]. These patterns of PS and CD47 expression allow for macrophages to identify CD47^+^ cells as self-structures [31,42]. In this case, accompanying high levels of PS are recognized as a mark of apoptosis, which triggers the phagocytosis of the cell and the release of anti-inflammatory signals that avoid autoimmunity against healthy tissues [49,50]. On the other hand, the absence of CD47 in a cell membrane reveals the presence of a potential infection [51,52]. Unlike the silent clearance of apoptotic host cells, phagocytosis of CD47^−^ cells is followed by the activation of the macrophage [53], and the secretion of pro-inflammatory signals that may lead to an innate immune response [54,55].

We postulate that the role of PS and CD47 in the phagocytosis of RBCs (as described in points E3 and E4) follows this general pattern. Young RBCs, like non-apoptotic host cells show high levels of CD47 and low levels of PS, which prevents their phagocytosis by macrophages. Among aged RBCs, those with high PS and low CD47 expression are comparable to apoptotic host cells, while those expressing very low levels of CD47 can be likened to foreign cells. Bearing these analogies in mind, we hypothesize the existence of two alternative pathways of RBC phagocytosis that entail different macrophage reactions. Specifically, we suggest that the pathway controlled by the balance between PS and CD47 (E3) is similar to the removal of apoptotic host cells. In particular, it does not trigger any immune response. By contrast, the phagocytosis of RBCs with very low CD47 expression (E4) might be analogous to the destruction of non-self agents by macrophages, and could provoke autoimmune reactions against host RBCs. In the remainder of this article, we will refer to both phagocytosis pathways as silent and immune, respectively.

The existence of an *ad hoc* mechanism to provoke autoimmunity may seem paradoxical. However, it has long been observed that auto-antibodies targeting host RBCs are usually present in the organism [56–58]. Anti-RBC antibodies are natural antibodies produced by B-1 cells [59,58]. Unlike antibodies from other B cell subsets, B-1 antibodies have anti-inflammatory effects, which minimize potential collateral damage to host tissues [60,61]. This might explain why anti-RBC auto-antibodies are usually innocuous [62] and only occasionally cause clinical disorders, known under the general term of autoimmune haemolytic anaemia [62]. On the other hand, natural antibodies are spontaneously produced in the absence of foreign antigens [56] so their specificity for RBCs cannot be explained as due to cross-reactivity of RBC epitopes and non-self structures encountered in previous infections. This raises the question of how these auto-antibodies are produced.

Our assumption of an immune pathway of RBC phagocytosis suggests a possible answer for this question. Macrophages of the MPS express MHC molecules, and can therefore act as antigen presenting cells [63,64]. We suggest that after phagocytizing RBCs with very low CD47 expression they would initiate an adaptive immune response, much like they do after phagocytizing foreign cells. As a matter of fact, it has been recently observed that removal of CD47 from self-RBCs suffices indeed to trigger immune responses in mice [65]. Nevertheless, since RBCs are not pathogens, macrophages of the MPS would recruit B-1 cells instead of more aggressive B cell types, leading to the production of non-inflammatory antibodies against host RBCs. The functional role of these anti-RBC auto-antibodies remains to be explained. In this respect, we will show in the following sections that anti-RBC autoimmunity, together with erythrophagocytosis and neocytolysis fit into a global, coherent model of RBC homeostasis. In order to do that, we will next state the previous conceptual model in mathematical terms.

### 2.1. Mathematical formalization of the conceptual model

As we have discussed above, quantitative dynamics of PS and CD47 seem to determine RBC lifespan (figure 1*d*–*f*). However, to the best of our knowledge, no such quantitative analysis is currently available in the literature. In the absence of empirical data, we will formulate a mathematical model that reproduces the qualitative features of PS and CD47 dynamics outlined in the previous section (see points E1 to E4). This model is based on the following assumptions:

(A1) The expression of ‘eat-me’ signals in the outer membrane of the RBC increases at a constant rate *β*.
(A2) The number of ‘don’t-eat-me’ signals decreases at a constant rate *α*. This results in an exponential decay, a behaviour that has been described for other RBC membrane proteins (e.g. [66,67]).
(A3) Two independent thresholds exist, denoted by *T*_*s*_ and *T*_*i*_, that trigger silent and immune phagocytosis pathways, respectively.

Assumptions A1 and A2 do not intend to account for the molecular mechanisms underlying the time evolution of membrane signals. Instead, they have been chosen for the sake of simplicity in order to show the relevance of signal dynamics in RBC homeostasis. Nevertheless, new data about PS and CD47 dynamics could be easily included in this approach by modifying assumptions A1 and A2. We will discuss the implications of this particular choice of assumptions in the last section of this article.

Denoting by *E*(*t*) and *D*(*t*) the number of ‘eat-me’ and ‘don’t-eat-me’ signals at time *t*, respectively, assumptions A1 and A2 can be stated in mathematical terms as follows:

2.1 $$\begin{array}{rl} D^{\prime }(t) & =-\alpha D(t)\\ \text{and}\;E^{\prime }(t) & =\beta , \end{array}\}$$

where *α* and *β* are positive parameters.

Integrating equations (2.1) we get an explicit expression for the dynamics of ‘eat-me’ and ‘don’t-eat-me’ signals:

2.2 $$\begin{array}{rl} D(t) & =D_{0}\;e^{-\alpha t}\\ \text{and}\;E(t) & =E_{0}+\beta t, \end{array}\}$$

where *E*_0_ and *D*_0_ are the amounts of ‘eat-me’ and ‘don’t-eat-me’ signals in the cell membrane at the birth of the RBC, respectively.

From assumption A3, it follows that conditions *E*(*t*_*s*_)−*D*(*t*_*s*_)=*T*_*s*_ and *D*(*t*_*i*_)=*T*_*i*_ define the times *t*_*s*_ and *t*_*i*_ at which the RBC is removed through the silent and immune phagocytosis pathways, respectively. Introducing the expressions of *E*(*t*) and *D*(*t*) given by equations (2.2) in these conditions, we get the following values for *t*_*i*_ and *t*_*s*_:

2.3 $$\begin{array}{rl} t_{i} & =\frac{1}{\alpha }\ln\left(\frac{D_{0}}{T_{i}}\right)\\ \text{and}\;t_{s} & =\frac{1}{\beta }(T_{s}-E_{0})+\frac{1}{\alpha }W\left(\frac{\alpha }{\beta }D_{0}\;e^{\alpha (E_{0}-T_{s})/\beta }\right), \end{array}\}$$

where *W*(⋅) is the product logarithm or Lambert *W* function.

Equations (2.3) define the conditions that dictate the fate of RBC. The cell is cleared through the silent pathway if *t*_*s*_<*t*_*i*_ and through the immune pathway otherwise. From equations (2.3), it follows that the timing of RBC phagocytosis, and hence its lifespan, is given by $L=\min(t_{i},t_{s})$.

## 3. A theoretical framework for red blood cell homeostasis

**Figure 2.**
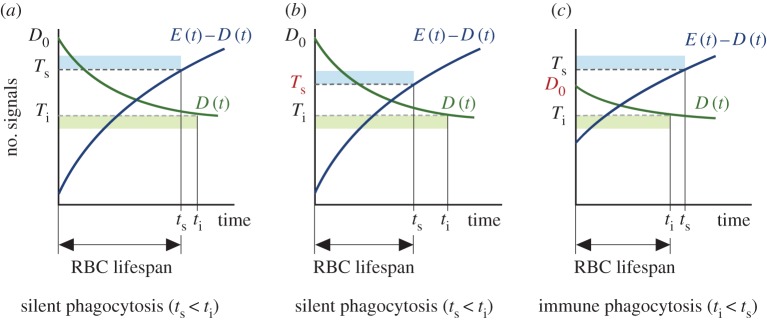
Results of the mathematical model of RBC lifespan determination. (*a*) The dynamics of membrane signals as defined by equations (2.1)–(2.3) satisfy the qualitative constraints imposed by empirical evidence (E1–E4). Both the lifespan of the cell and how it is phagocytized (i.e. through the silent or the immune pathway) depend on the particular values of the model parameters. In this case, the difference between ‘eat-me’ and ‘don’t-eat-me’ signals is the first to reach its critical threshold (*T*_*s*_), so that this cell is destroyed through the silent pathway at time *t*_*s*_ (which sets its lifespan). (*b*) Changing the silent threshold (parameter *T*_*s*_ in the model) shortens the lifespan of the cell, but not the phagocytosis pathway. (*c*) By contrast, lower CD47 expression at the birth of the cell (parameter *D*_0_) both shortens the lifespan of the cell and changes the condition that triggers its phagocytosis (from silent to immune).

From equations (2.1) to (2.3), ‘eat-me’ and ‘don’t-eat-me’ signals can be viewed as defining a cellular algorithm whose execution in the membrane of each RBC determines both its fate (i.e. if it is removed through the silent or the immune pathway) and its lifespan. In this section, we will show that this algorithm provides a coherent, integrative view of RBC homeostasis. According to equations (2.3), RBC fate and lifespan are unambiguously defined by the specific values of six parameters. Roughly speaking, these parameters represent the amount of membrane signals at the birth of the cell (*D*_0_ and *E*_0_), the rates of change of these signals (*α* and *β*), and the thresholds that elicit the phagocytosis of RBCs (*T*_*s*_ and *T*_*i*_). Variations in any of these features result in changes in either the lifespan of the cell or the phagocytosis pathway leading to its destruction (figure 2). Bearing this fact in mind, we will next enumerate a series of biological mechanisms that could be used by the organism to modulate RBC lifespan, and discuss their consequences on RBC homeostasis.

### 3.1. Effects of oxidative stress on red blood cell lifespan

As we noted above, OS causes the accumulation of defects in the cytosol and membrane of RBCs, increasing the probability of malfunction and even of cell lysis in the blood. In extreme cases, this may induce a severe clinical condition known as haemolysis [68]. RBCs showing signs of oxidative damage should therefore be removed from the circulation in order to minimize the risk of haemolysis. It has been suggested that the level of PS expression is one of those signs, since higher levels of OS are accompanied by higher rates of PS externalization [34,35,69,70].

**Figure 3.**
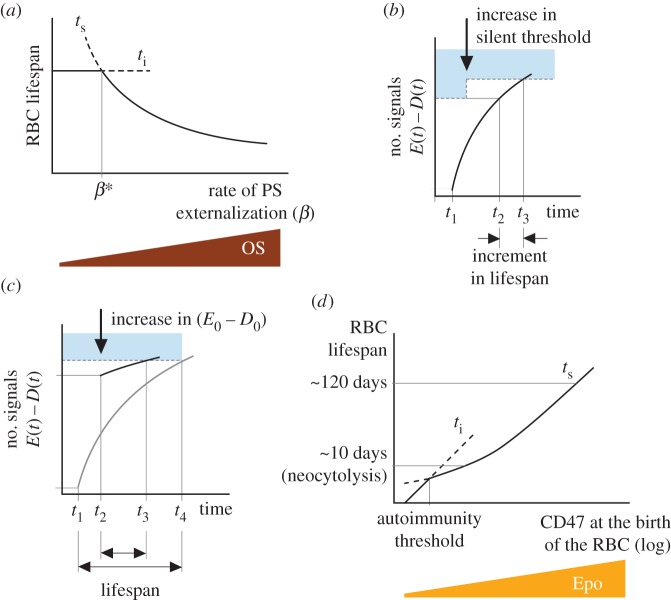
Potential mechanisms of RBC lifespan modulation. (*a*) Higher levels of oxidative stress are associated with higher rates of PS externalization. In agreement with empirical data, the model predicts an inverse correlation between the degree of OS and RBC lifespan. If the rate of PS externalization is above a critical value (β*) the curve of *t*_*s*_ (time to reach the silent threshold) is below the curve of *t*_*i*_ (time to attain the immune threshold). This implies that for high values of OS (*β*>β*) RBCs are phagocytized through the silent pathway. Only if *β*<β* are RBCs destroyed through the immune pathway, which can lead to anti-RBC autoimmunity. (*b*) Time evolution of the difference between ‘eat-me’ and ‘don’t-eat-me’ signals in the membrane of an RBC formed at time *t*_1_. The difference between membrane signals should reach the silent threshold at time *t*_2_, thereby causing the phagocytosis of the cell. Increasing the silent threshold delays the phagocytosis of the RBC until *t*_3_, thus extending its lifespan. (*c*) *Neocytolysis*. The figure shows the dynamics of the difference between ‘eat-me’ and ‘don’t-eat-me’ signals in the membrane of two RBCs that differ in the expression of membrane signals at birth. The first cell, formed at time *t*_1_, is phagocytized at time *t*_4_ after a normal lifespan. The second cell, born at time *t*_2_>*t*_1_ with a larger difference between ‘eat-me’ and ‘don’t-eat-me’ signals in its membrane attains the silent threshold much faster, so it is destroyed at time *t*_3_, before the first cell and after a much shorter lifespan. (*d*) According to our model, the lifespan of each RBC is directly correlated with the level of CD47 expressed in its membrane when it is formed. Low values of CD47 expression could explain short lifespans observed during neocytolysis. Furthermore, if the initial amount of CD47 falls below a critical level (the autoimmunity threshold), the immune phagocytosis occurs before the silent pathway (*t*_*i*_<*t*_*s*_). In this case, macrophages of the MPS phagocytize RBCs after very short lifespans and initiate anti-RBC autoimmune responses.

PS exposure in response to OS is not a passive process. Instead, it seems to be mediated by cytoplasmic RBC proteins [27], suggesting that RBCs are able to accelerate the rate of PS externalization in case of oxidative damage. From this observation, we can deduce that higher values of parameter *β* (rate of PS externalization) correspond to RBCs exposed to higher levels of OS (see equations (2.1)). In agreement with empirical observations, this condition shortens RBC lifespan [20,70] (figure 3). From the perspective of our model, accelerated PS exposure in response to OS can be interpreted as an active mechanism to minimize the risk of RBC lysis in the blood. By increasing the rate of PS translocation, an RBC would hasten its phagocytosis through the silent pathway. The cell would therefore be removed from the circulation before attaining a critical level of oxidative damage that might compromise its function or even its physical viability.

### 3.2. Recovery of red blood cell homeostasis after haemorrhages

According to equations (2.3), tuning the silent phagocytosis threshold provides another mechanism to modulate RBC lifespan. This threshold is defined as the difference between ‘eat-me’ and ‘don’t-eat-me’ signals that triggers the silent phagocytosis pathway in macrophages of the MPS. Hence, from a mechanistic point of view, tuning this parameter amounts to modulating the sensitivity of macrophages to RBC signals. Increasing the silent phagocytosis threshold delays phagocytosis and extends RBC lifespan (equations (2.1) and figure 3*b*). Each day added to mean RBC lifespan prevents the destruction of 10^11^ cells (around 1% of the total population), which is equivalent to the daily production of RBCs in normal conditions.

A significant fall in the number of RBCs after a haemorrhage may produce a deficit of oxygen in the tissues. The subsequent rise in the levels of Epo in the blood [71] eventually restores the population of RBCs and the equilibrium of oxygen. However, given that this process involves the differentiation of precursor cells it can take a few days to take the population back to its original size. Increasing the silent threshold could buffer cell loss and help to maintain the supply of oxygen until Epo-mediated recovery of the population is completed. In this regard, we remark that empirical evidence suggests that phagocytosis of macrophages of the MPS is indeed suppressed after haemorrhages [72]. Furthermore, macrophages are equipped with Epo receptors [73], implying that the tuning of the silent phagocytosis threshold might be directly controlled by the levels of plasma Epo. Once the equilibrium of oxygen is recovered, the levels of Epo would return to normal values, restoring both the silent threshold and RBC lifespan.

### 3.3. Neocytolysis and erythrophagocytosis

Neocytolysis and erythrophagocytosis are currently considered as alternative mechanisms of RBC removal [3,13]. In particular, it is implicitly assumed that erythrophagocytosis is the default pathway of destruction of senescent RBCs during normal homeostasis, while neocytolysis is somehow triggered by decreased levels of Epo [9,11]. Such drops of Epo occur, in particular, whenever oxygen availability in the tissues is above physiological needs. For instance, people descending to sea level after a period of acclimation to high altitudes move from lower to higher partial pressures of atmospheric oxygen. In this situation, the population of RBCs is larger than needed to ensure the supply of oxygen to the tissues, and contracts to a new equilibrium size through the selective destruction of younger RBCs [74]. The mechanisms underlying the switch from erythrophagocytosis to neocytolysis remain poorly understood [9,70].

In this work, we suggest that neocytolysis and erythrophagocytosis should not be considered as independent mechanisms, but as alternative outcomes of the algorithm of RBC lifespan determination. Specifically, both processes can be explained as caused by different patterns of PS and CD47 expression in the membrane of newly formed RBCs. Figure 3*c* compares the lifespan of two RBCs that differ in the amount of membrane signals at birth. The RBC with the bigger difference between PS and CD47 expression is the first one to reach the silent phagocytosis threshold, even if it is born later. Moreover, this cell is destroyed after a short lifespan, while the other is spared and will only be removed after reaching the usual RBC lifespan. These features are precisely what defines neocytolysis. Therefore, according to our model, neocytolysis occurs if RBCs formed under lower levels of Epo are born with more PS or less CD47 in their outer membrane. Empirical evidence points to the latter, since young RBCs show lower levels of CD47 and similar levels of PS (when compared with older cells) in people descending to sea level after acclimation to high altitude [12].

This result suggests that the transition from erythrophagocytosis to neocytolysis does not require a switch between alternative mechanisms of RBC destruction. Instead, the lifespan of RBCs can vary in a continuum that ranges from 10 days during neocytolysis, to 80 days in newborns and 120 days in adult humans, depending on the level of PS and/or CD47 at the birth of the cells. In order to illustrate the main point of this work, and for the sake of simplicity, we will continue our discussion assuming that Epo only affects CD47 expression in newly formed RBCs (figure 3*d*). Similar arguments could be drawn if Epo also determined initial PS levels.

### 3.4. Autoimmune responses in red blood cell homeostasis

Neocytolysis reduces the number of RBCs when oxygen supply exceeds the demands of body tissues [9,11]. We suggest that autoimmunity could provide a complementary mechanism to accelerate the contraction of the RBC population in such circumstances. According to the model, anti-RBC autoimmune responses emerge from the same process that leads to neocytolysis, namely, the reduction in CD47 expression in newly formed RBCs. If the population of RBCs is still larger than required after neocytolysis-driven contraction, the levels of Epo continue to drop. In consequence, newly formed RBCs express progressively less CD47 in their membranes (figure 3*d*). RBCs whose initial levels of CD47 expression falls beyond a critical point (labelled as the autoimmune threshold) are phagocytized through the immune pathway (figure 3*d*). The ensuing production of natural auto-antibodies would foster the death of other RBCs, further contracting the population.

The view of anti-RBC autoimmune responses as a homeostatic mechanism is supported by the fact that natural antibodies do not target all circulating RBCs, which might result in a massive and uncontrolled loss of cells. Instead, they are directed against specific epitopes usually expressed in aged RBCs and absent in young cells [57]. Moreover, RBCs of any age are also protected from the action of auto-antibodies by CD47, which is known to inhibit the phagocytosis of opsonized cells [75,28]. On the other hand, the protection provided by CD47 is dose-dependent [75], implying that the destruction of an individual RBC through this antibody-mediated pathway depends on both its levels of CD47 and the concentration of antibodies present in the blood. For this reason, only those RBCs with high CD47 expression survive in the course of more aggressive responses. Therefore, the intensity of the autoimmune response (i.e. the amount of auto-antibodies produced) determines the cohorts of RBC that are destroyed, and hence the extent of the reduction in the number of cells.

In normal conditions, autoimmunity-driven contraction of the population should eventually restore physiological levels of oxygen. Under the assumptions of our model, the subsequent rise in Epo would increase CD47 expression in new RBCs, arresting the production of anti-RBC antibodies (figure 3*d*). Further increases of initial CD47 would also interrupt neocytolysis and restore RBC lifespan to normal values observed in erythrophagocytosis. Therefore, Epo-dependent regulation of CD47 in new RBCs creates a switch between silent and immune phagocytosis and makes both neocytolysis and homeostatic autoimmunity reversible processes.

Our model also suggests that anti-RBC responses are only triggered if levels of OS are sufficiently low (figure 3*a*). Assuming the homeostatic nature of autoimmunity, this result can be understood as preventing the production of auto-antibodies in conditions of severe OS. Under these circumstances, oxidative damage can cause the abnormal destruction of many RBCs, making unlikely the need for anti-RBCs antibodies to remove an excess of cells.

### 3.5. The role of Epo in red blood cell lifespan determination

The role of Epo in RBC production and its relationship to oxygen homeostasis are well established in the literature [3]. It has been hypothesized that Epo could also control the onset of neocytolysis by modulating the interaction between macrophages of the MPS and young circulating RBCs [11]. The theoretical model presented in this work supports this hypothesis by suggesting an explicit mechanism that links Epo to RBC lifespan determination. Moreover, this model suggests that neocytolysis can be understood as a particular manifestation of a more general function of Epo as determinant of RBC destruction. This function would consist in setting RBC lifespan by adjusting the phagocytosis thresholds and the levels of CD47 expression in newly formed cells. If proven correct, this model would explain a variety of RBC responses to changes in oxygen supply to the tissues.

**Figure 4.**
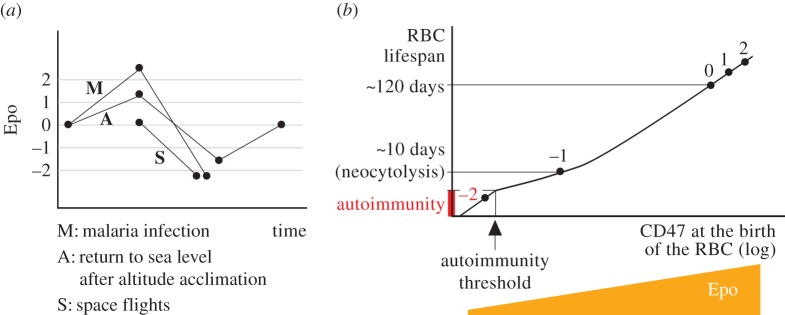
A theoretical model for the relationship between RBC lifespan and oxygen homeostasis. (*a*) Acclimation to environments with different partial pressures of oxygen, or clinical conditions that involve massive RBC loss such as malaria entail sharp fluctuations in the levels of plasma Epo (see text for references). (*b*) We hypothesize that Epo controls CD47 expression in newly formed RBCs, which in turn sets their expected lifespan (see equations (2.3)). In normal conditions both Epo and oxygen levels are at equilibrium, and mean RBC lifespan is around 120 days (0). Any variation in Epo, independently of its cause, changes the amount of CD47 in newly formed RBCs and hence its lifespan. From this perspective, a pronounced decrease in Epo suffices to account for the onset of neocytolysis observed in people returning to sea level after high-altitude acclimation or in malaria patients (labelled as −1 in the figure). Further drops of Epo can lead to autoimmunity (labelled as −2), which could explain the presence of auto-antibodies against host RBCs in malaria patients or in astronauts after space flights. See the text for further details.

For instance, people descending to sea level after high-altitude acclimation show sharp fluctuations of Epo owing to altitude-related changes in the partial pressure of oxygen. Epo increases during acclimation to higher altitudes, and falls after returning to sea levels, attaining lower values than those found before altitude acclimation [12,70] (figure 4*a*). Similar Epo dynamics have been described in malaria patients. The destruction of RBCs by *Plasmodium* parasites during the first stages of malaria causes a deficit of oxygen in the tissues, and a subsequent increase of Epo [76,77]. By contrast, later stages of the infection are usually associated with insufficient production of Epo [77–79]. Epo also falls if partial pressure of oxygen increases, e.g. during spaceflights or in the return to sea level after high-altitude acclimation [80,9].

We postulate that any drop in Epo is expected to exert similar effects on RBC lifespan independently of its cause. Within the framework of our model, these effects range from neocytolysis to the initiation of homeostatic autoimmunity (figure 4*b*). As a matter of fact, both neocytolysis and strong anti-RBC responses have been described in astronauts after space flights [9,74]. As for malaria infections, both *Plasmodium falciparum* and *P. vivax* infections cause the abnormal removal of an important number of non-parasitized cells (npRBCs) [81,82]. In some patients of severe malaria-derived anaemia, the destruction of npRBCs can even continue long after the infection has been cleared (see [83] and references therein). Therefore, malarial anaemia cannot be explained simply by the direct destruction of infected RBCs alone. Both selective death of young non-parasitized RBCs [84–86] and the presence of anti-RBC antibodies [87] suggest that neocytolysis and homeostatic autoimmunity might play a major role in the development of anaemia during malaria. In this situation, the anomalous drop of Epo that characterizes latter stages of malaria infection would be erroneously perceived by the organism as caused by an excess of circulating RBCs. The ensuing normal homeostatic mechanisms (neocytolysis and homeostatic autoimmunity) triggered in abnormal conditions of oxygen availability would lead to anomalous reductions in the population of RBCs.

## 4. Discussion

The consumption of oxygen by the organism is highly variable owing to factors such as circadian metabolic rhythms, the intensity of physical activity or even fluctuations in ambient temperature [88,89]. In consequence, homeostatic mechanisms must continuously adjust the balance between RBC production and destruction to maintain an appropriate number of RBCs. The control of RBC production by Epo is well described in the literature [3]. By contrast, many questions about RBC destruction remain largely unanswered. In particular, no universally accepted explanation of the mechanisms underlying changes in RBC lifespan is available as yet.

A substantial body of evidence points to PS and CD47 as key determinants of RBC phagocytosis [26–31]. In this work, we postulate that quantitate aspects of these dynamics explain how RBC lifespan variations are related to oxygen homeostasis. This statement is based on two main assumptions. First, that the pattern of PS and CD47 expression changes during the life of the cell, as evidenced by differences between young and aged RBCs [34–38]. Second, that the conditions that trigger RBC phagocytosis as described in the literature (see points E3 and E4 above) differ in the subsequent behaviour they elicit on macrophages of the MPS. Specifically, we postulate that the phagocytosis of RBCs with very low levels of CD47 provokes immune responses against host RBCs.

The nature of this work is necessarily speculative owing to the lack of published data about the actual dynamics of CD47 and PS in the membrane of RBCs. We have modelled plausible dynamics that satisfy the constraints imposed by available evidence. The exponential decay proposed for CD47 has actually been described for other molecules present in RBCs [66,67]. As for the increase of PS externalization observed in ageing cells, we have assumed that it occurs at a constant rate for the sake of simplicity. A different mathematical formalization of the model would involve a different set of parameters, suggesting perhaps other mechanisms of RBC lifespan modulation. In any case, the conceptual model that emerges from published evidence (outlined in figure 1) is independent of any particular mathematical formulation. From this conceptual model, PS and CD47 constitute a molecular clock that sets the timing of RBC phagocytosis. RBC lifespan should be determined by the time it takes for these signals to satisfy one of the two conditions that trigger the phagocytosis of the cell.

A mathematical version of this conceptual model suggests several mechanisms that might modulate RBC lifespan. First, changes in CD47 expression in newly formed RBCs could account for differences in lifespan observed in erythrophagocytosis and neocytolysis, as well as for the origin and function of anti-RBC autoimmunity. We remark that none of these processes is explicitly implemented in the equations of the model. Instead, they emerge as alternative outcomes of the same algorithm of lifespan determination for different values of initial CD47 expression at the birth of the cell. Second, by controlling macrophage phagocytic activity, Epo levels might continuously adjust the value of the phagocytosis thresholds, thus fine-tuning the lifespan of circulating RBCs. Finally, higher levels of OS might shorten RBC lifespan by accelerating the rate of PS exposure in the outer membrane of the cell. These mechanisms are independent and might be acting simultaneously to determine RBC lifespan. In this respect, it has been recently suggested that hypoxia-induced factors (HIFs) might be involved in shortening RBC lifespan during neocytolysis [70]. The effect of HIF would be related to lower catalase activity in young RBCs formed in hypoxia. Under this assumption, such young RBCs would be more susceptible to OS in case of a rise in oxygen availability, which would translate into higher rates of PS externalization. From the perspective of our model, this would imply that parameter *β* takes higher values in RBCs formed during hypoxia. At the same time, the amount of CD47 in new RBCs could be modulated by Epo depending on the levels of oxygen. The combined effects of accelerated PS expression and lower CD47 expression would result in shortened RBC lifespan and, eventually, in the production of anti-RBC auto-antibodies. In turn, autoimmunity and neocytolysis would rapidly contract the population whenever oxygen supply is above physiological needs.

Further research is needed to unveil all the mechanisms underlying RBC lifespan determination. However, irrespectively of their ultimate causes, variations in RBC lifespan play a central role in the ability of the organism to modulate the rate of RBC destruction. Specifically, if all human RBCs lived 120 days, then the temporal pattern of cell destruction would just reproduce the pattern of formation of new RBCs with a delay of 120 days. Extending mean lifespan beyond 120 days lowers the rate of cell destruction and enlarges the number of RBCs in the blood. Conversely, the phagocytosis of RBCs under 120 days of age contracts the population by increasing the rate of cell destruction. Therefore, it is clear that any theory intending to explain RBC homeostasis should explicitly address the question of how RBC lifespan is determined. The conceptual model introduced in this work constitutes a first step towards the development of such a theory. We believe that this model will improve our understanding of how RBC homeostasis is maintained in normal circumstances and how its imbalance can lead to pathology.
